# Solution of Iodoform

**Published:** 1873-12

**Authors:** Louis Elsberg


Solution of Iodoform—*By Louis Elsberg, M.D.*—The conclusions drawn by the authors from their experiments are:

1. To employ iodoform in the chrystalline state;
2. To make the solution in a *red* glass flask by simple agitation;
3. To use the following proportions:

Crystalized Iodoform, 1 gramme;
Ether, (60 deg. Baume), 4 grammes.

I had a solution prepared with Squibb's ether, and find that it possesses all the advantages of iodoform in powder for local applications without its disadvantages. The smarting which the ether may be expected to produce upon the mucous membrane is momentary only, so that the application becomes really painless. Its beneficial effects surpass my expectations.—*Phil. Med. Times.*

